# Multidimensional evaluation of quality differences for *Dendrobium officinale* stems grown under different cultivation environments based on widely targeted metabolomics, network pharmacology, molecular docking, and cell experiments

**DOI:** 10.3389/fpls.2025.1501545

**Published:** 2025-03-28

**Authors:** Yingyue Hou, Guangying Du, Jing Li, Pei Liu, Jinqiang Zhang

**Affiliations:** ^1^ School of Information Engineering, Guizhou University of Traditional Chinese Medicine, Guiyang, China; ^2^ Resource Institute for Chinese and Ethnic Materia Medica, Guizhou University of Traditional Chinese Medicine, Guiyang, China

**Keywords:** *Dendrobium officinale*, metabolomics, network pharmacology, quality evaluation, molecular docking

## Abstract

**Introduction:**

*Dendrobium officinale* is an endangered perennial epiphytic herbaceous plant. In the Chinese Pharmacopoeia, the dried stems of *D. officinale* are used medicinally and are commonly utilized as a medicinal and food homologous product. Notable variations in the quality of *D. officinale* stems are observed across different cultivation environments; however, the underlying mechanisms remain unclear.

**Methods:**

Metabolites in *D. officinale* stems grown in stone epiphytic, tree epiphytic, and greenhouse environments were identified using UPLC-MS/MS-based widely targeted metabolomics. Differential metabolites from stems grown in different cultivation environments were selected for studies on quality differences. Network pharmacology was employed to investigate the core targets of these differential metabolites, and molecular docking validation was conducted with these metabolites to identify quality markers. Finally, a combination of network pharmacology and *in vitro* experimental results was used to explore the reasons behind the differences in therapeutic effects of *D. officinale* stems grown in various cultivation environments.

**Results:**

A total of 1929 primary and secondary metabolites were identified. Compared to the tree epiphytic and greenhouse environments, 58 primary and secondary metabolites were up-regulated in the stone epiphytic environment. Among these, 7 amino acids and their derivatives were exclusively found as up-regulated primary metabolites, while 18 flavonoids constituted the main up-regulated secondary metabolites. The binding affinities of the 18 flavonoids to the core targets (MAOA and TNF) were superior to those of other up-regulated metabolites, and they can be utilized in quality difference studies, particularly nicotiflorin and isoquercitrin. Stems grown in the stone epiphytic environment showed a superior protective effect on chronic atrophic gastritis cells compared to the other two environments. This was associated with increased binding of differential metabolites to targets such as MAOA and TNF and decreased binding to targets such as SRC and PTGS2.

**Discussion:**

The composition and content of metabolites in *D. officinale* stems are influenced by the cultivation environment, which in turn affects the therapeutic effects of the stems. The change of the target preference could be the reason for the difference in drug efficacy. This study introduces a novel approach for distinguishing the quality of *D. officinale* stems grown under different cultivation environments and exploring the variations in their therapeutic effects.

## 1 Introduction

*Dendrobium* is one of the three largest genera in the Orchidaceae family (Schuiteman, 2011). *Dendrobium officinale* Kimura et Migo, regarded as the most valuable species within the genus *Dendrobium*, is a perennial epiphytic plant commonly found across Southeast and South Asia. In the Chinese Pharmacopoeia (2020 edition), only the dried stems of *D. officinale* are used, with reported effects that include benefiting the stomach, generating fluids, nourishing *Yin*, and clearing heat (National Pharmacopoeia Commission, 2020). Modern pharmacological studies have also demonstrated that *D. officinale* stems are rich in active compounds (Yang et al., 2023a) and are widely utilized as a functional food (Luo et al., 2017). Notably, on November 9, 2023, the National Health Commission of China officially included *D. officinale* stems in the “Catalogue of Substances Traditionally Used as Both Food and Herbal Medicine,” affirming their long-term safety for consumption (National Health Commission et al., 2023).

Numerous studies have shown that *D. officinale* stems possess various health benefits, including the improvement of gastrointestinal mucosa injury (Yang et al., 2020), anti-colitis effects (Liang et al., 2018), anticancer properties (Liu et al., 2023), anti-diabetes activity (Lv et al., 2023), immune regulation (Dong et al., 2022), anti-liver injury effects (Lei et al., 2019), and protection against metabolic hypertension (Li et al., 2021). These therapeutic effects are primarily attributed to the rich bioactive compounds found in the plant, such as polysaccharides, flavonoids, alkaloids, and free amino acids (Xu et al., 2022; Wu et al., 2020). In the Chinese Pharmacopoeia, moisture content, total ash, alcohol-soluble extract, polysaccharide content, and mannose content serve as quality standards for the dried stems of *D. officinale* (National Pharmacopoeia Commission, 2020). As a result, most current quality evaluations and pharmacological activity studies of *D. officinale* focus on the polysaccharides found in its stems. However, the chemical complexity and diversity of plants further suggest that relying on a single compound is insufficient to comprehensively assess the quality of medicinal plants (Luo et al., 2024; Qaderi et al., 2023). Secondary metabolites also play a vital role in the pharmacological activities of medicinal plants (Strzemski et al., 2019). Flavonoids, the main secondary metabolites in *D. officinale* stems, are significant contributors to its physiological activity (Zuo et al., 2020). These compounds exhibit a range of pharmacological properties, including antioxidant and hypoglycemic effects (Ross and Kasum, 2002; Luo et al., 2023). Therefore, it is necessary to conduct a comprehensive analysis of the primary and secondary metabolites of *D. officinale* stems.

Due to long-term overharvesting, the wild resources of *D. officinale* are nearing extinction. Currently, the market primarily relies on artificially cultivated *D. officinale*, with three main cultivation methods: Stone epiphytic cultivation (on black limestone with carbonate rock as the parent rock), Tree epiphytic cultivation (on the bark of Schima superba trees), and Greenhouse cultivation (on the bark of pine trees) (Cheng et al., 2019). Notably, classical texts emphasize that *D. officinale* stems grown in stone epiphytic cultivation environment are regarded as having the highest quality (Zheng et al., 2024). It is well known that the composition and content of secondary metabolites in plants are influenced by their growth environment (Hornyák et al., 2022). Research has shown that *D. officinale* stems grown in wild stone epiphytic cultivation environment have higher polysaccharide, total flavonoid, and total alkaloid content than those from greenhouse environment (Yuan et al., 2020). On the other hand, *D. officinale* stems grown in stone epiphytic cultivation have significantly higher levels of secondary metabolites compared to those grown in tree epiphytic and greenhouse environments (Yang et al., 2023b). Therefore, there are significant differences in the quality of *D. officinale* stems grown in these three cultivation environments, with those grown in stone epiphytic cultivation environment potentially having higher quality. However, how to fully evaluate the quality of *D. officinale* stems in different cultivation environments is still a problem. The identification of suitable quality markers for controlling the quality of medicinal plants has become widely recognized as essential (Dong et al., 2020; Zeng et al., 2023). The widely targeted metabolomics, also known as second-generation targeted metabolomics, analysis based on liquid chromatography-tandem mass spectrometry (UPLC-MS/MS) is a fast and reliable method for detecting plant metabolites (Fraga et al., 2010). It overcomes the limitations of targeted metabolomics, which focuses on a narrow range of compounds, and addresses the shortcomings of untargeted metabolomics, such as reduced accuracy in both qualitative identification and quantitative analysis due to the lack of standardized references (Li et al., 2022). Based on UPLC-MS/MS, screening appropriate metabolites for quality evaluation of medicinal plants represents a viable direction (Tong et al., 2021).

On the other hand, the mechanism of action of a metabolite or the entire crude extract of *D. officinale* stems on its target is not yet fully understood. A promising direction for further research involves studying the mechanism of action by analyzing the interactions between multiple components and their target preferences (Zeng et al., 2023). By integrating metabolomics data with network pharmacology, a compound-target-disease network can be constructed, allowing for the exploration of the material basis and molecular mechanisms of traditional Chinese medicine in treating diseases from a systematic and holistic perspective. However, traditional network pharmacology analysis typically only quantifies compound-target-disease correlations without evaluating their strength (Li and Zhang, 2013). Therefore, in this study, the predicted target scores and Protein-Protein Interaction (PPI) network scores based on the metabolites of *D. officinale* stems were weighted to identify important targets.

This study collected samples of *D. officinale* stems from three cultivation environments: tree epiphytic cultivation, stone epiphytic cultivation, and greenhouse cultivation. Ultra Performance Liquid Chromatography-Tandem Mass Spectrometry (UPLC-MS/MS) technology was employed to analyze the metabolites of *D. officinale* stems, and weighted network pharmacology was constructed to investigate the bioactive components and mechanisms of action of *D. officinale* stems. Eighteen flavonoid types were selected as references for cultivating high-quality *D. officinale* stems, and the target preference mechanism of *D. officinale* stems was preliminarily explored. Additionally, the advantages and disadvantages of *D. officinale* stems under different cultivation environments for treating liver cancer and chronic atrophic gastritis were investigated through *in vitro* experiments. The experimental process is shown in **Supplementary Figure S1**. The research findings provide a foundational basis for the rational assessment of *D. officinale* stems quality across different cultivation environments and hold significant scientific importance for the high-quality development of the *D. officinale* forest understory ecological planting industry.

## 2 Materials and methods

### 2.1 Sampling and preparing

**Figure 1:**
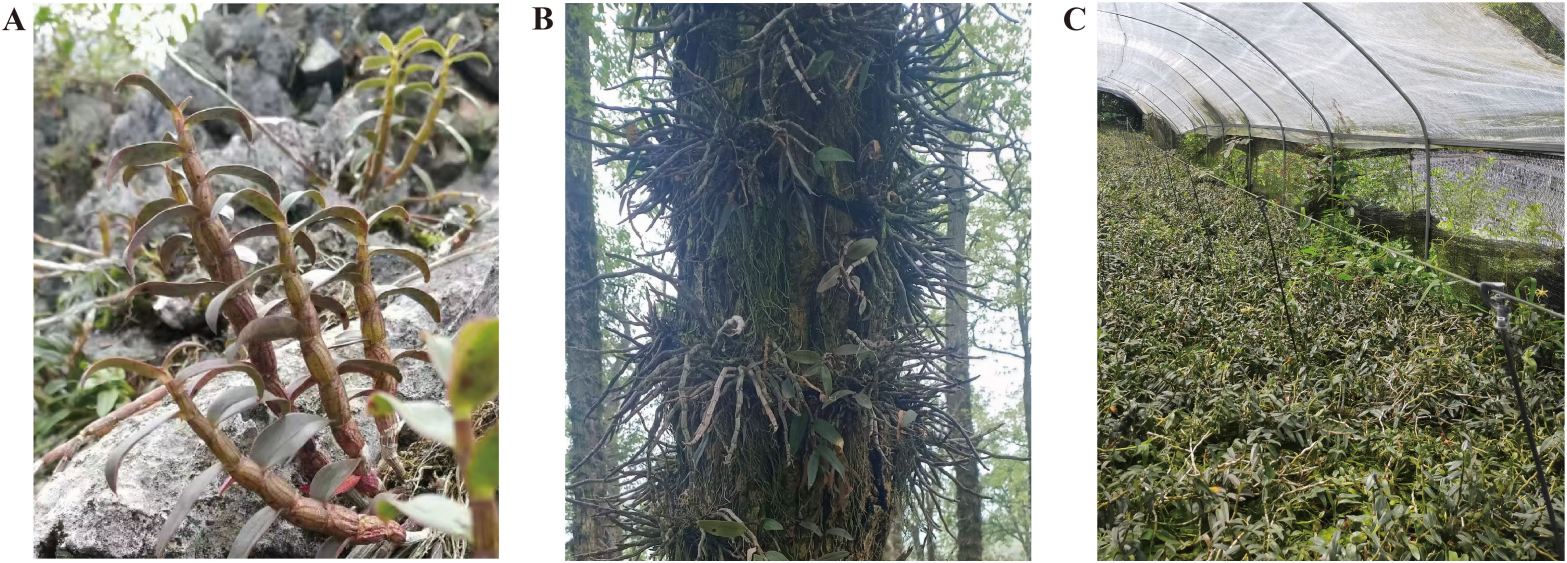
The three growth environments of *D. officinale*: **(A)** Stone epiphytic culture environment in the wild. **(B)** Tree epiphytic culture environment in the wild. **(C)** Greenhouse culture environment.

Fresh stems (2-year-old) of *D. officinale* were collected from Anlong County in Southwest Guizhou, China, in April 2023. Details of the sample collection are shown in **Table 1**. To ensure the accuracy and representativeness of the collected samples, nine stems were combined to form one sample, with three replicate samples per group. A total of nine sample groups were classified as SEC 1-3 (stone epiphytic culture environment, **Figure 1A**), TEC 1-3 (tree epiphytic culture environment, **Figure 1B**), and GC 1-3 (greenhouse culture environment, **Figure 1C**). The plant samples were identified by Guangying Du. The samples were placed into sampling bags and stored in a dry ice bucket on site. Upon returning to the laboratory, the samples were stored at -80°C for later use.

**Table 1:** The information of the selected samples.

Cultivation Environments	Epiphytic Substrates	Time of Harvesting	Growing Years	Elevation (m)	Climate Type
Tree epiphytic cultivation (TEC)	*Schima superba*	202304	2 years old	1135.6	Subtropical monsoon climate
Stone epiphytic cultivation (SEC)	Limestone	202304	2 years old	1152.2	Subtropical monsoon climate
Greenhouse cultivation (GC)	Pine bark	202304	2 years old	1119.5	–

Using vacuum freeze-drying technology, place the biological samples in a lyophilizer (Scientz-100F), then grinding (30 Hz, 1.5 min) the samples to powder form by using a grinder (MM 400, Retsch). Next, weigh 50 mg of sample powder using an electronic balance (MS105Dμ) and add 1200 μL of -20°C pre-cooled 70% methanolic aqueous internal standard extract (less than 50 mg added at the rate of 1200 μL extractant per 50 mg sample). Vortex once every 30 min for 30 sec, for a total of 6 times. After centrifugation (rotation speed 12000 rpm, 3 min), the supernatant was aspirated, and the sample was filtered through a microporous membrane (0.22 μm pore size) and stored in the injection vial for UPLC-MS/MS analysis.

### 2.2 Metabolite identification and quantification

#### 2.2.1 Widely targeted metabolomics

The widely targeted metabolomics employed in this study is based on the multiple reaction monitoring (MRM) mode of UPLC-MS/MS. UPLC: Utilizes ultra-high pressure and small particle size chromatographic columns to achieve efficient separation of metabolites, thereby minimizing co-elution of compounds; MS/MS: Employs a triple quadrupole mass spectrometer (QQQ), where the first quadrupole (Q1) screens for parent ions of target metabolites, the second quadrupole (Q2) fragments these parent ions into characteristic daughter ions through collision-induced dissociation (CID), and the third quadrupole (Q3) selectively monitors specific daughter ions; MRM mode: Based on the known molecular weights (m/z) of metabolites, specific parent ions are screened in Q1. In Q3, only the characteristic daughter ions generated from the fragmentation of parent ions via CID are monitored (Chen et al., 2013; Dunn et al., 2013).

#### 2.2.2 UPLC conditions

The sample extracts were analyzed using an UPLC-ESI-MS/MS system (UPLC, ExionLC™ AD, *https://sciex.com.cn/* ) and Tandem mass spectrometry system ( *https://sciex.com.cn/* ). The analytical conditions were as follows, UPLC: column, Agilent SB-C18 (1.8 µm, 2.1 mm * 100 mm); The mobile phase was consisted of solvent A, pure water with 0.1% formic acid, and solvent B, acetonitrile with 0.1% formic acid. Sample measurements were performed with a gradient program that employed the starting conditions of 95% A, 5% B. Within 9 min, a linear gradient to 5% A, 95% B was programmed, and a composition of 5% A, 95% B was kept for 1 min. Subsequently, a composition of 95% A, 5.0% B was adjusted within 1.1 min and kept for 2.9 min. The flow velocity was set as 0.35 mL per minute; The column oven was set to 40°C; The injection volume was 2 μL. The effluent was alternatively connected to an ESI-triple quadrupole-linear ion trap (QTRAP)-MS.

#### 2.2.3 ESI-Q TRAP-MS/MS

The ESI source operation parameters were as follows: source temperature 500°C; ion spray voltage (IS) 5500 V (positive ion mode)/-4500 V (negative ion mode); ion source gas I (GSI), gas II (GSII), curtain gas (CUR) were set at 50, 60, and 25 psi, respectively; the collision-activated dissociation (CAD) was high. QQQ scans were acquired as MRM experiments with collision gas (nitrogen) set to medium. DP (declustering potential) and CE (collision energy) for individual MRM transitions was done with further DP and CE optimization. A specific set of MRM transitions were monitored for each period according to the metabolites eluted within this period.

#### 2.2.4 Qualitative and quantitative analysis of metabolites

Mass spectrometry data were processed using Analyst 1.6.3 software. The total ion current (TIC), which represents the sum of all ion intensities in the mass spectrum plotted over time, was analyzed for the quality control (QC) samples from the mixed group. Additionally, multi-peak chromatograms from metabolite detection in MRM mode (ion chromatography spectrum for multi-substance extraction, XIC) were evaluated. The x-axis denotes the retention time (Rt) for metabolite detection, while the y-axis represents ion current intensity, measured in counts per second (CPS). The labels “N” and “P” correspond to negative and positive ion modes, respectively. The TIC and MRM chromatograms are presented in **Supplementary Figures S2**-**Supplementary Figures S3**. Qualitative and quantitative metabolite analysis was conducted using a local metabolic database (Metware database, Wuhan Metware Biotechnology Co., Ltd., Wuhan, 430070, China). The MRM multi-peak detection plot shows the metabolites identified in the samples, with each chromatographic peak assigned a distinct color corresponding to a specific metabolite. A triple quadrupole mass spectrometer was used to screen characteristic ions for each metabolite, and the signal intensity (CPS) of these ions was recorded by the detector. Raw mass spectrometry data files were processed using MultiQuant software for chromatographic peak integration and correction. The peak area (Area) of each chromatographic peak was used to represent the relative abundance of the corresponding metabolite. All integrated chromatographic peak area data were exported and saved for further analysis. To evaluate differences in metabolite content across samples, chromatographic peaks for each metabolite were corrected based on retention time and peak shape information. This approach ensured accurate qualitative and quantitative results for all detected metabolites. The comparison results are shown in **Supplementary Figure S4**.

### 2.3 Statistical analysis and differential metabolites selected

#### 2.3.1 Principal component analysis

Unsupervised principal component analysis (PCA) was performed by statistics function prcomp within R ( *www.r-project.org* ). The data was unit variance scaled before unsupervised PCA.

#### 2.3.2 Hierarchical cluster analysis and pearson correlation coefficients

The HCA (hierarchical cluster analysis) results of samples and metabolites were presented as heatmaps with dendrograms, while pearson correlation coefficients (PCC) between samples were calculated by the cor function in R and presented as only heatmaps. Both HCA and PCC were carried out by R package ComplexHeatmap. For HCA, normalized signal intensities of metabolites (unit variance scaling) are visualized as a color spectrum.

#### 2.3.3 Differential metabolites selected

The characteristics of metabolomics data are “high-dimensional and massive,” necessitating the integration of both univariate and multivariate statistical analysis methods. These methods should be applied from multiple perspectives according to the data’s properties to accurately identify differential metabolites. Univariate statistical analysis methods encompass hypothesis testing and fold change (FC) analysis. Multivariate statistical analysis methods include PCA, orthogonal partial least squares discriminant analysis (OPLS-DA), among others. Based on the variable importance in projection (VIP) values obtained from the OPLS-DA model (with biological replicates ≥ 3), preliminary screening of metabolites differing among different varieties or tissues can be conducted (Zeng et al., 2024). Simultaneously, the P-value/FDR obtained from univariate analysis (Li et al., 2023) or FC value (Sun et al., 2020b) can be combined to further filter out differential metabolites. In this study, for two-group analysis, differential metabolites were identified based on VIP > 1, *p* < 0.01, and |Log_2_FC| ≥ 1.0. VIP values were extracted from the OPLS-DA results, which also included score plots and permutation plots, generated using the R package MetaboAnalystR. Data were log-transformed and mean-centered before OPLS-DA. To avoid overfitting, a permutation test with 200 permutations was performed.

#### 2.3.4 KEGG annotation and enrichment analysis

Identified metabolites were annotated using KEGG Compound database ( *http://www.kegg.jp/kegg/compound/* ), annotated metabolites were then mapped to KEGG Pathway database ( *http://www.kegg.jp/kegg/pathway.html* ). Pathways with significantly regulated metabolites mapped to were then fed into MSEA (metabolite sets enrichment analysis), their significance was determined by hypergeometric test’s p-values.

### 2.4 Weighted network pharmacology analysis

#### 2.4.1 Collection of differential metabolite targets and disease targets

The SMILES format files of differential metabolites were retrieved from the PubChem database ( *https://pubchem.ncbi.nlm.nih.gov/* ). The SMILES format files of the differential metabolites were input into the Swiss Target Prediction database ( *https://www.swisstargetprediction.ch* ) to predict the corresponding targets of these compounds. Targets with a probability greater than 0.12 were selected and summarized. Through a literature review, three diseases were identified where *D. officinale* stems may have therapeutic potential: type 2 diabetes, Alzheimer’s disease, and liver cancer. Relevant disease targets were then searched for and downloaded from the GeneCards database ( *https://www.genecards.org/* ).

#### 2.4.2 Weighted-targeted network analysis

The targets from the Swiss Target Prediction database were input into the protein-protein interaction network (PPI) of the STRING database ( *https://www.string-db.org/* ), with the interaction score set to greater than 0.4 (**Supplementary Figure S5**). Combined with the results of the Swiss Target Prediction and STRING analysis, a weighted-target network was constructed by Gephi 0.10.1, which can be downloaded from *https://gephi.org/users/download/* . The target degree was calculated using Cytoscape_v3.10.0 (**Supplementary Table S1**). To perform KEGG enrichment analysis on the targets of differential metabolites, the targets were entered into the DAVID database, with the identifier set to “SYMBOL” and the species selected as “Homo sapiens”. After completing the analysis, the results were further visualized using a bioinformatics website ( *https://www.bioinformatics.com.cn/* ).

### 2.5 Collection of compound structures and receptor protein

The two-dimensional (2D) or three-dimensional (3D) structures of the compounds were downloaded from the PubChem database and saved in SDF format. The 2D structures of some compounds were provided by Wuhan Mateweier Biotechnology Co., Ltd. These compound structures are used as ligands. The SDF files were then converted to PDB format using Chem3D 2022, which served as small molecule ligands. The 3D structures of MAOA (Monoamine oxidase A, PBD ID: 2Z5X), TNF (Tumor Necrosis Factor, PDB ID: 1NCF), SRC (SRC protein tyrosine kinase, PDB ID: 3D7T), and PTGS2 (Prostaglandin Endoperoxide Synthase 2, PDB ID: 5IKV) were obtained from the RCSB PDB database ( *https://www.rcsb.org/* ).

### 2.6 Molecular docking simulation

PyMOL 2.6 was used to remove water molecules and ligands from receptor proteins. AutoDockTools 1.5.6 was used to choose the torsions of ligands and add polar hydrogens of protein receptor molecules. Molecular docking simulation was performed using AutoDock Vina 1.1.2 (Liu et al., 2024; Zeng et al., 2023) (AutoDock Vina – molecular docking and virtual screening program ( *https://vina.scripps.edu/* )). The accuracy of AutoDock Vina molecular docking simulations is approximately 80% (Trott and Olson, 2010; Eberhardt et al., 2021). In this study, each molecular docking was conducted over 20 times to improve reliability, with lower docking scores indicating more stable binding. The result files were visualized using PyMOL 2.6.

### 2.7 *In vitro* experiments

#### 2.7.1 Liver cancer test

Human liver cancer cells (HepG2) were seeded uniformly at a density of 5000 cells per well in a 96-well plate and incubated for 24 hours in a 37°C, 5% CO_2_ incubator. The ethanol extract of *D. officinale* stems was diluted to concentrations of 800 μg mL^-1^, 400 μg mL^-1^, 200 μg mL^-1^, 100 μg mL^-1^, and 50 μg mL^-1^, with 100 μL of each concentration added per well. The control group received 100 μL of MEM medium only. The plates were then incubated for 24 hours. After incubation, the culture medium was discarded, and 100 μL of 10% CCK-8 solution was added to each well. The plates were incubated for an additional hour, and absorbance at 450 nm was measured using an enzyme-linked immunosorbent assay (ELISA) reader.

#### 2.7.2 Chronic atrophic gastritis test

Human gastric mucosal cells (GES-1) were seeded uniformly at a density of 4000 cells per well in a 96-well plate and incubated for 24 hours in a 37°C, 5% CO_2_ incubator. The ethanol extract of *D. officinale* stems was diluted to concentrations of 100 μg mL^-1^, 50 μg mL^-1^, 25 μg mL^-1^, 12.5 μg mL^-1^, and 6.25 μg mL^-1^, with 100 μL of each concentration added per well. The control group and model group received 100 μL of DMEM medium only. The plates were incubated for 24 hours, after which the culture medium was discarded. To induce a chronic atrophic gastritis (CAG) model, 100 μL of 80 μmol/L 1-Methyl-3-nitro-1-nitrosoguanidine (MNNG) solution was added to each well, except for the control group, which received 100 μL of DMEM culture medium only. The plates were incubated for another 24 hours. Following incubation, the culture medium was discarded, and 100 μL of 10% CCK-8 solution was added to each well. The plates were incubated for 1 hour, and absorbance at 450 nm was measured using an enzyme-linked immunosorbent assay (ELISA) reader.

## 3 Results

### 3.1 Multivariate analysis of metabolomes

#### 3.1.1 Widely targeted metabolome profiles

**Figure 2:**
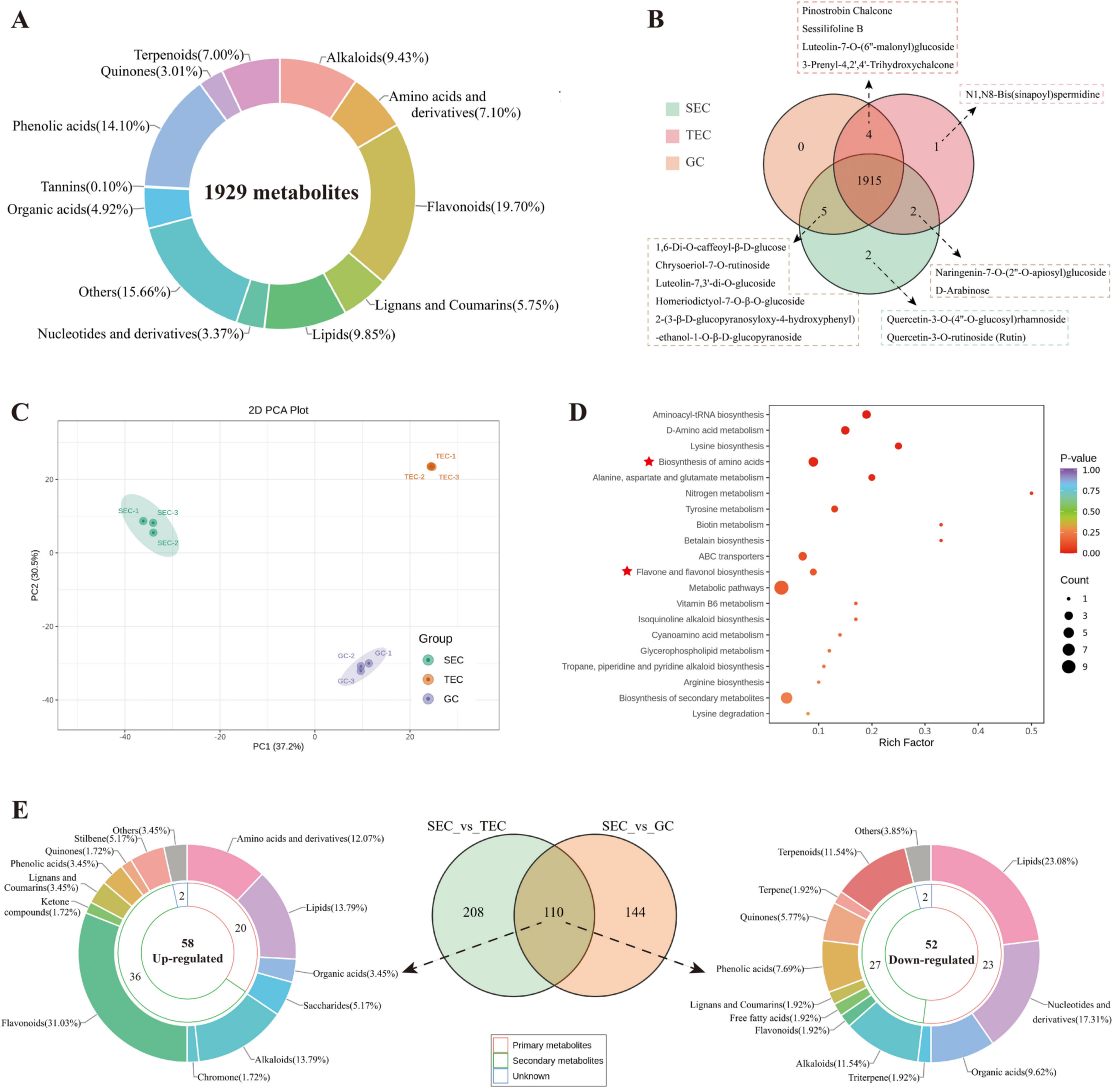
Widely targeted metabolome profiles of *D. officinale* stems in three cultivation environments. **(A)** Classification circular diagram of total metabolites in *D. officinale* stems from the three cultivation environments. **(B)** Venn diagram of total metabolites in *D. officinale* stems from the three cultivation environments. **(C)** PCA results of metabolites in *D. officinale* stems from the three cultivation environments. **(D)** KEGG (metabolics) enrichment analysis of up-regulated differential metabolites in *D. officinale* stems in different cultivation environments. **(E)** Venn diagram of differential metabolites in *D. officinale* stems and classification circular diagram of up-regulated and down-regulated metabolites.

The UPLC-MS/MS results revealed that the types of metabolites present in *D. officinale* stems from the three different cultivation environments were generally similar. A total of 1929 metabolites were identified in the stems of *D. officinale* across the three cultivation environments (**Supplementary Table S2**, **Figure 2A**). These included 182 alkaloids (9.43%), 137 amino acids and derivatives (7.1%), 380 flavonoids (19.7%), 111 lignans and coumarins (5.75%), 190 lipids (9.85%), 65 nucleotides and derivatives (3.37%), 95 organic acids (4.92%), 302 other compounds (15.66%), 272 phenolic acids (14.1%), 58 quinones (3.01%), 2 tannins (0.1%), and 135 terpenes (7.0%). Notably, quercetin-3-O-(4’’-O-glucosyl) rhamnoside and quercetin-3-O-rutinoside (rutin) were flavonoids uniquely detected in the stems of *D. officinale* cultivated in the SEC environment. Additionally, N1,N8-bis(sinapoyl)spermidine was detected exclusively in the stems of *D. officinale* cultivated in the TEC environment (**Figure 2B**). These 1,929 metabolites were further classified (**Supplementary Figure S6**).

Principal component analysis (PCA) revealed significant differences in the metabolites of *D. officinale* stems from the three cultivation environments (**Figure 2C**). When comparing the metabolite contents of *D. officinale* stems, 110 metabolites exhibited significant differences across the three environments (SEC_vs_other, |Log_2_FC| ≥ 1.0, *p* < 0.01, VIP > 1), with 58 metabolites being up-regulated and 52 metabolites down-regulated (**Supplementary Table S3**). These 110 differential metabolites were further categorized into primary and secondary metabolites (**Figure 2E**). Among them, 20 primary metabolites and 36 secondary metabolites were up-regulated in the SEC environment, while 23 primary metabolites and 27 secondary metabolites were down-regulated. Flavonoids (18/36) were the major category of up-regulated secondary metabolites, including dihydrocharcone-4’-O-glucoside, phloretin-4’-O-glucoside (Trilobatin), 6-C-methylkaempferol-3-glucoside, chrysoeriol-5-O-glucoside, chrysoeriol-7-O-(6’’-malonyl)glucoside, diosmetin-7-O-galactoside, diosmetin-7-O-glucoside, diosmetin-8-C-(2’’-O-arabinosyl)glucoside, isohyperoside, isorhamnetin-3-O-(6’’-malonyl)glucoside-7-O-glucoside, kaempferol-3-O-(6’’’’-malonyl)sophorotrioside, kaempferol-3-O-(6’’-O-acetyl)glucoside, kaempferol-3-O-rutinoside (nicotiflorin), myricetin-3-O-β-D-glucoside, quercetin-3-O-glucoside (isoquercitrin), quercetin-7-O-glucoside, hesperetin-5-O-glucoside, and homeriodictyol-7-O-β-O-glucoside. Additionally, amino acids and their derivatives (7/20) were found exclusively in the up-regulated primary metabolites, including (2S)-2-amino-4-methyl-4-pentenoic acid, cycloleucine, L-asparagine, L-glutamine, L-lysine, N-methyl-L-proline, and γ-glutamyl-L-valine.

#### 3.1.2 Metabolic pathways

**Figure 3:**
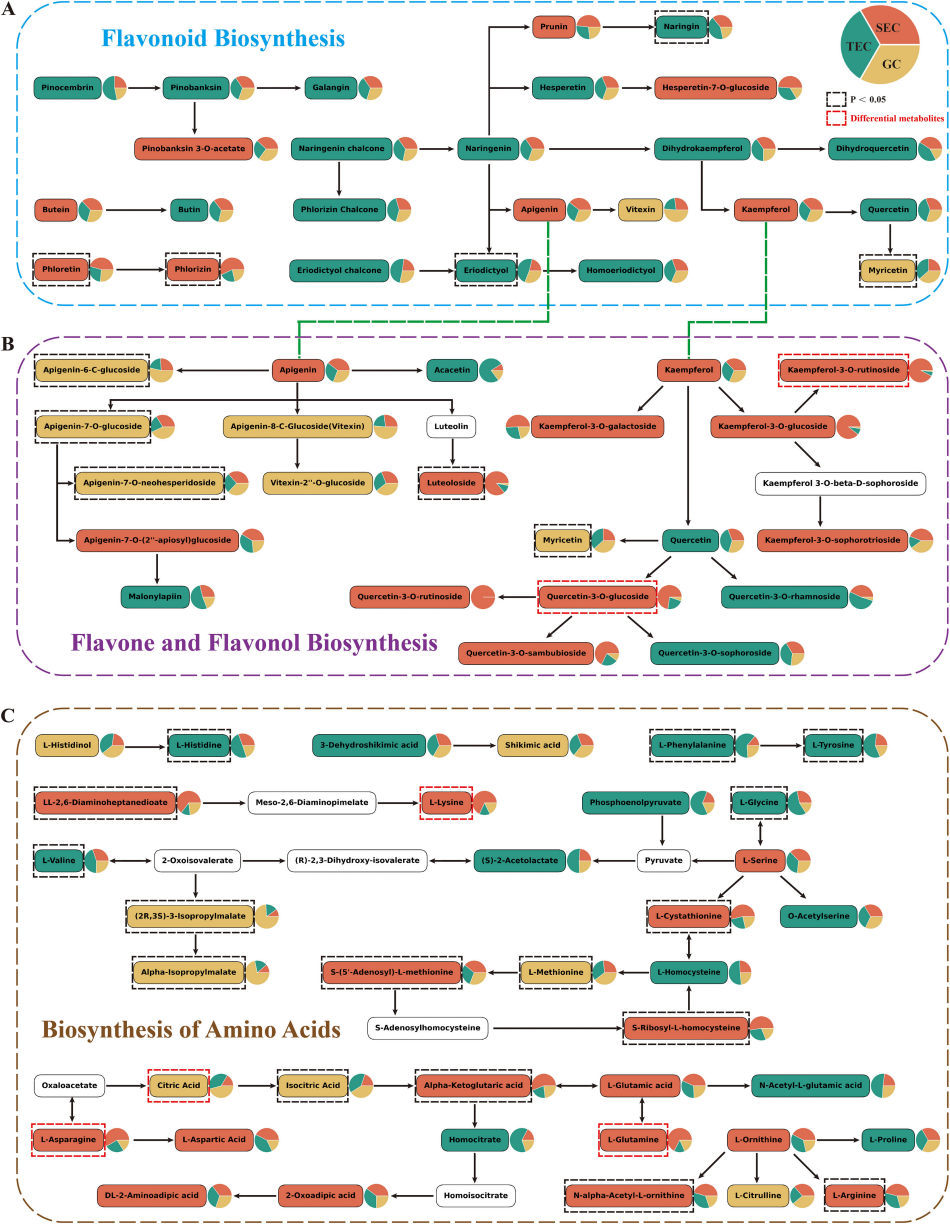
Metabolic pathways of *D. officinale* stems. The background colors of metabolites correspond to the group with higher content, and the red dashed box indicates differential metabolites. **(A)** Flavonoid Biosynthesis. **(B)** Flavone and Flavonol Biosynthesis. **(C)** Biosynthesis of Amino Acids.

KEGG (metabolics) enrichment analysis of up-regulated differential metabolites (**Figure 2D**) revealed that up-regulated primary metabolites were predominantly enriched in the amino acid biosynthesis pathway, while up-regulated secondary metabolites were primarily associated with the flavone and flavonol biosynthesis pathways. To further investigate, this study conducted heatmap analysis of the metabolic pathways of flavonoids, specifically the flavonoid biosynthetic pathway (ko00941, **Figure 3A**), the flavone and flavonol biosynthetic pathway (ko00944, **Figure 3B**), and the amino acid biosynthesis pathway (ko01230, **Figure 3C**).

The major metabolites in the flavonoid pathway of *D. officinale* stems included flavones, flavonols, flavanones, and chalcones. Interestingly, the results showed that 15 metabolites linked to the flavonoid biosynthesis pathway were more abundant in the TEC environment, while 8 metabolites had higher levels in the SEC environment, with phloretin and phlorizin showing a significant increase (*p* < 0.05) (**Figure 3A**). Additionally, 11 metabolites associated with the flavone and flavonol biosynthesis pathway were found to be more abundant in SEC compared to TEC (5 metabolites) and GC (6 metabolites). These included kaempferol and its derivatives (kaempferol-3-O-rutinoside [nicotiflorin], kaempferol-3-O-galactoside, kaempferol-3-O-glucoside, kaempferol-3-O-sophorotrioside) as well as some quercetin derivatives (quercetin-3-O-glucoside [isoquercitrin], quercetin-3-O-rutinoside, quercetin-3-O-sambubioside). Notably, nicotiflorin and isoquercitrin were among the up-regulated differential metabolites. In contrast, the content of apigenin derivatives was higher in the GC environment (**Figure 3B**). Moreover, 16 metabolites associated with the amino acid biosynthesis pathway were more abundant in SEC compared to TEC (13 metabolites) and GC (8 metabolites), with 7 metabolites—LL-2,6-diaminoheptanedioate, L-cystathionine, S-(5’-adenosyl)-L-methionine, S-ribosyl-L-homocysteine, α-ketoglutaric acid, N-α-acetyl-L-ornithine, and L-arginine—showing a significant increase (*p* < 0.05). L-lysine, L-glutamine, and L-asparagine were among the up-regulated differential metabolites, while citric acid was a down-regulated differential metabolite (**Figure 3C**).

In conclusion, the flavone and flavonol biosynthesis pathway and the amino acid biosynthesis pathway are more active in the stems of *D. officinale* cultivated in the SEC environment compared to the other two cultivation environments.

#### 3.1.3 KEGG (genes) enrichment analysis of targets of differential metabolites

**Figure 4:**
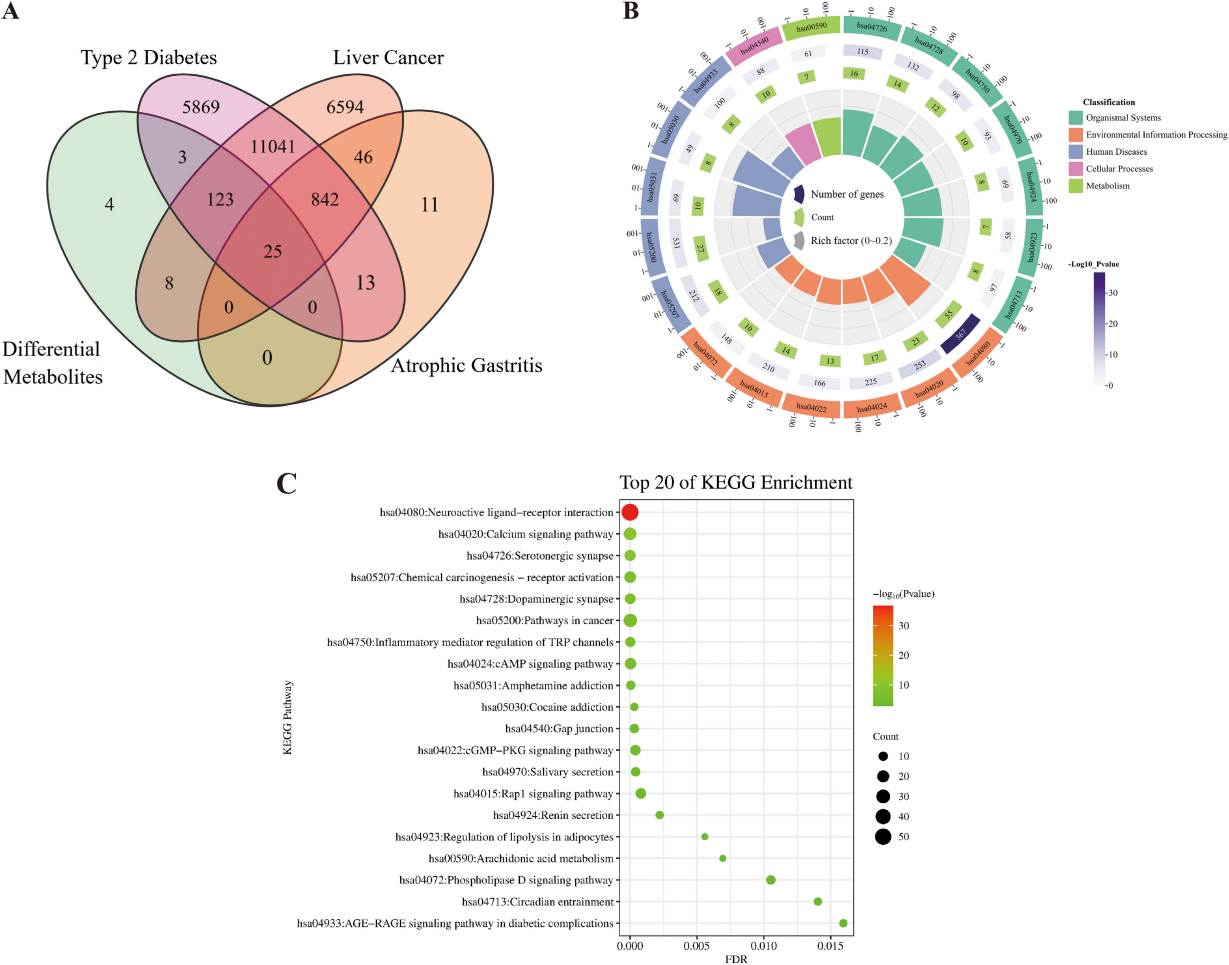
Target prediction and functional annotation in the stems of *D. officinale* across three cultivation environments. **(A)** Venn diagram showing the overlap of targets for differential metabolites in *D. officinale* stems across three cultivation environments and three diseases (Liver cancer, Type 2 diabetes, Chronic atrophic gastritis). **(B)** Circular plot of KEGG enrichment results. **(C)** Bubble chart of KEGG enrichment results.

The differential metabolite analysis revealed that, compared to the TEC and GC environments, 58 metabolites were up-regulated and 52 metabolites were down-regulated in the SEC environment. To explore the reasons behind the therapeutic differences in *D. officinale* stems grown under different cultivation environments, network pharmacology was applied to analyze the 58 up-regulated and 52 down-regulated metabolites. According to the Swiss Target Prediction database, a total of 163 targets (Probability ≥ 0.12) were identified for the differential metabolites (SEC_vs_other) in *D. officinale* stems, consisting of 110 targets for up-regulated metabolites and 66 targets for down-regulated metabolites. A Venn diagram was created to show the overlap between the three disease targets and the differential metabolite targets, revealing 25 common targets (**Figure 4A**).

All targets of the differential metabolites were selected for KEGG (genes) enrichment analysis. The KEGG pathway enrichment analysis identified 51 pathways (**Supplementary Table S4**), with the top 20 pathways selected based on *p* values to generate KEGG enrichment circle plots and KEGG enrichment bubble plots. The analysis indicated that the targets of differential metabolites were mainly concentrated in organismal systems, environmental information processing, and human diseases. Significant pathways included the serotonergic synapse (organismal systems, hsa04726), neuroactive ligand-receptor interaction (environmental information processing, hsa04080), amphetamine addiction (human diseases, hsa05031), and cocaine addiction (human diseases, hsa05030). Other important pathways involved the calcium signaling pathway (environmental information processing, hsa04020), chemical carcinogenesis receptor activation (human diseases, hsa05207), dopaminergic synapse (organismal systems, hsa04728), inflammatory mediator regulation of TRP channels (organismal systems, hsa04750), and pathways in cancer (human diseases, hsa05200) (**Figures 4B, C**).

### 3.2 Weighted network pharmacology-based analysis of differential metabolites

#### 3.2.1 Constructing a weighted network diagram for target points

**Figure 5:**
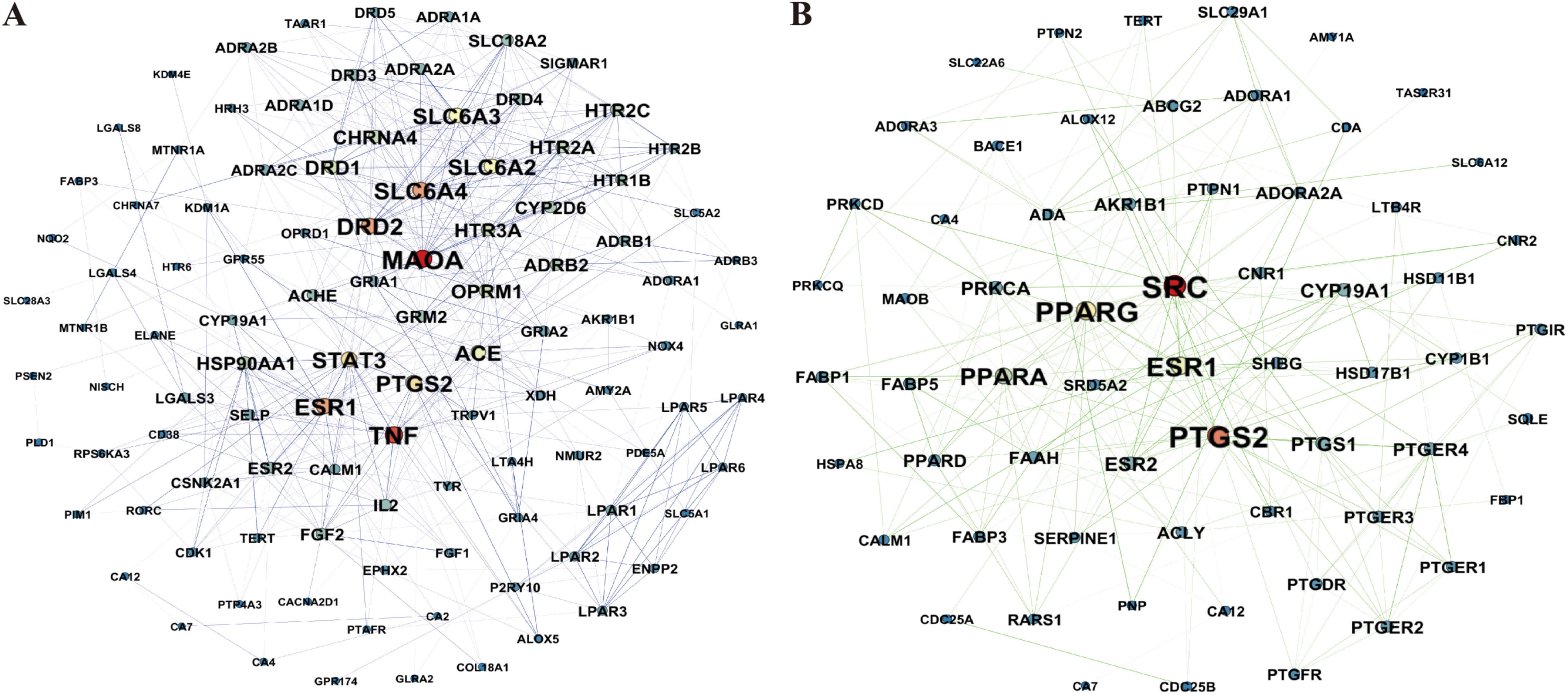
Weighted network pharmacology analysis of differential metabolites in the stems of *D. officinale* grown in a stone epiphytic culture environment compared to other cultivation environments. **(A)** Weighted network pharmacology analysis of up-regulated metabolites in the stems of *D. officinale* grown in a stone epiphytic culture environment *vs*. other environments. **(B)** Weighted network pharmacology analysis of down-regulated metabolites in the stems of *D. officinale* grown in a stone epiphytic culture environment *vs*. other environments.

The study identified 110 targets for up-regulated metabolites and 66 targets for down-regulated metabolites. To determine the core targets, a weighted analysis of the targets was conducted. Among the up-regulated differential metabolite (SEC_vs_other) targets, Monoamine Oxidase A (MAOA) and Tumor Necrosis Factor (TNF) emerged as the highest-weighted targets. In contrast, SRC protein tyrosine kinase (SRC) and Prostaglandin Endoperoxide Synthase 2 (PTGS2) were the highest-weighted targets among the down-regulated differential metabolite targets (**Figure 5**, **Supplementary Table S1**). This suggests that the benefits of the SEC environment, compared to the TEC and GC environments, might be attributed to alterations in the differential metabolite content in SEC, which increases binding to MAOA receptors and TNF, while reducing binding to SRC and PTGS2.

#### 3.2.2 Molecular docking

**Figure 6:**
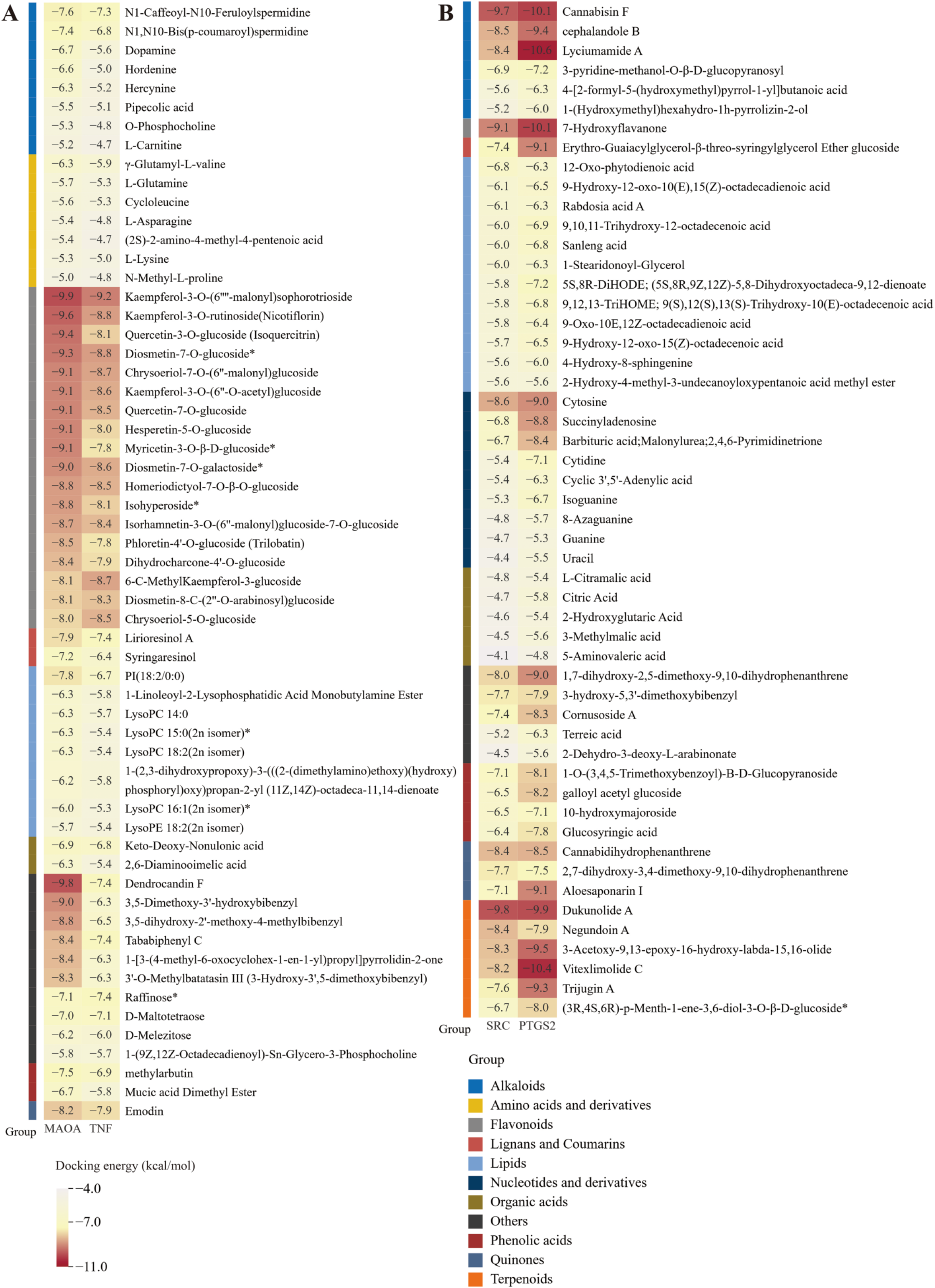
Molecular docking score results. **(A)** Molecular docking score results of up-regulated differential metabolites with MAOA and TNF. **(B)** Molecular docking score results of down-regulated differential metabolites with SRC and PTGS2.

**Figure 7:**
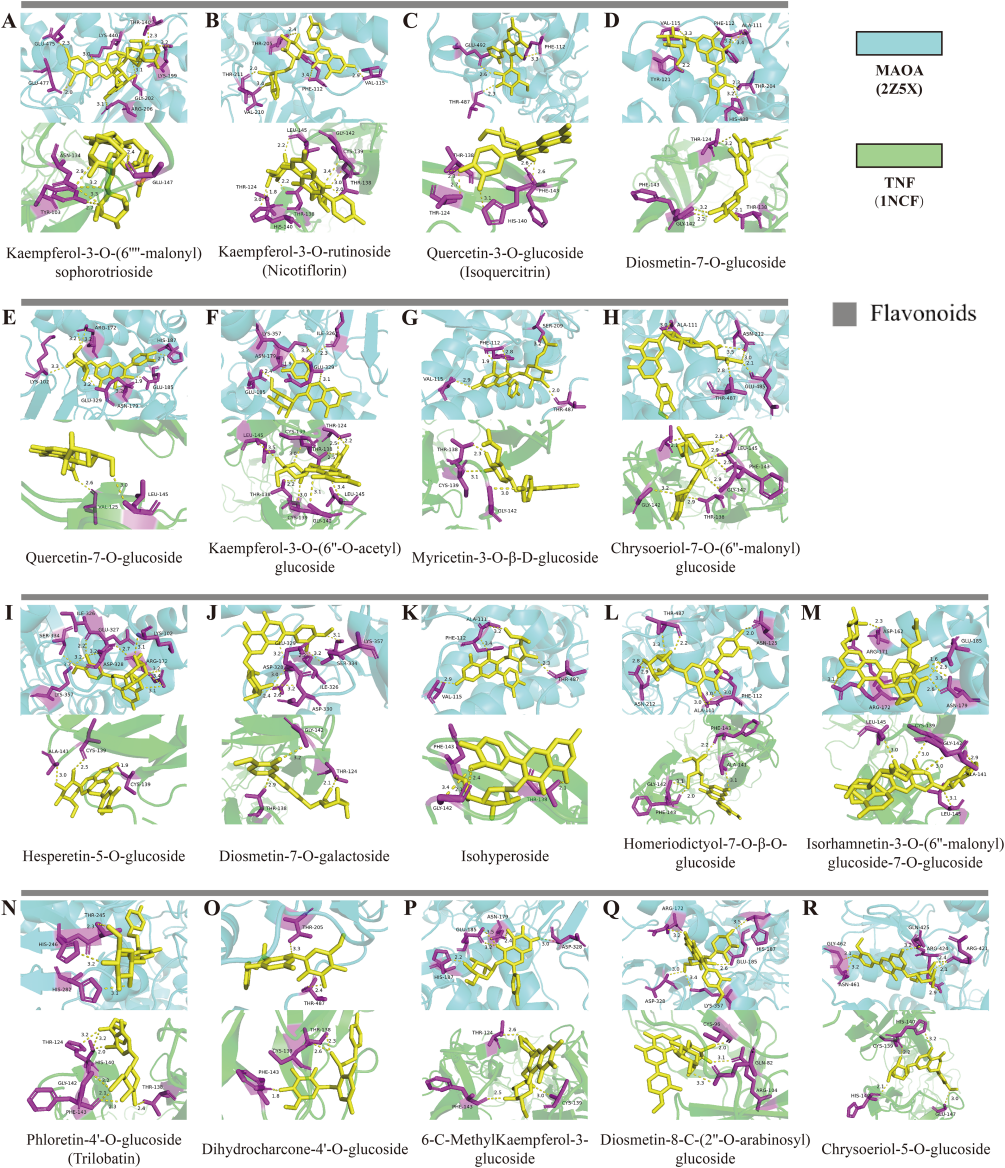
Molecular docking results of up-regulated flavonoids in *D. officinale* stems from different cultivation environments. **(A)** Predicted binding mode of Kaempferol-3-O-(6’’-malonyl) sophorotrioside with MAOA and TNF. **(B)** Predicted binding mode of Kaempferol-3-O-rutinoside (Nicotiflorin) with MAOA and TNF. **(C)** Predicted binding mode of Quercetin-3-O-glucoside (Isoquercitrin) with MAOA and TNF. **(D)** Predicted binding mode of Diosmetin-7-O-glucoside with MAOA and TNF. **(E)** Predicted binding mode of Quercetin-7-O-glucoside with MAOA and TNF. **(F)** Predicted binding mode of Kaempferol-3-O-(6’’-O-acetyl)glucoside with MAOA and TNF. **(G)** Predicted binding mode of Myricetin-3-O-β-D -glucoside with MAOA and TNF. **(H)** Predicted binding mode of Chrysoeriol-7-O-(6’’-malonyl)glucoside with MAOA and TNF. **(I)** Predicted binding mode of Hesperetin-5-O-glucoside with MAOA and TNF. **(J)** Predicted binding mode of Diosmetin-7-O -galactoside with MAOA and TNF. **(K)** Predicted binding mode of Isohyperoside with MAOA and TNF. **(L)** Predicted binding mode of Homeriodictyol-7-O-β-O-glucoside with MAOA and TNF. **(M)** Predicted binding mode of Isorhamnetin-3-O-(6’’-malonyl)glucoside-7-O-glucoside with MAOA and TNF. **(N)** Predicted binding mode of Phloretin-4’-O-glucoside (Trilobatin) with MAOA and TNF. **(O)** Predicted binding mode of Dihydrocharcone-4’-O-glucoside with MAOA and TNF. **(P)** Predicted binding mode of 6-C-MethylKaempferol-3-glucoside with MAOA and TNF.**(Q)** Predicted binding mode of Diosmetin-8-C-(2’’-O-arabinosyl)glucoside with MAOA and TNF. **(R)** Predicted binding mode of Chrysoeriol-5-O-glucoside with MAOA and TNF.

Compared to the TEC and GC environments, the SEC environment led to the up-regulation of 58 differential metabolites and the down-regulation of 52 differential metabolites. To examine the binding capacity between these differential metabolites and core targets, the 58 up-regulated metabolites were docked with MAOA and TNF (**Figure 6A**), while the 52 down-regulated metabolites were docked with SRC and PTGS2 (**Figure 6B**). A docking energy value below -4.25 kcal·mol^-^¹ suggests some binding activity between the molecules, a value below -5.0 kcal·mol^-^¹ indicates good binding affinity, and a value below -7.0 kcal·mol^-^¹ denotes strong binding affinity. The results showed that the up-regulated metabolites displayed binding activity with both MAOA and TNF, especially flavonoids, which exhibited docking energy values lower than -8.0 kcal·mol^-^¹ with MAOA and lower than -7.8 kcal·mol^-^¹ with TNF. Among these, kaempferol-3-O-(6’’-malonyl) sophorotrioside demonstrated the lowest docking energy values with these two proteins (-9.9 and -9.2 kcal·mol^-^¹, respectively). Nicotiflorin and isoquercitrin, key components in the flavone and flavonol biosynthetic pathways (**Figure 3B**), also exhibited strong binding capacity. While amino acids and their derivatives showed some binding activity with MAOA and TNF (all below -4.8 kcal·mol^-^¹), they were not as potent. These 18 flavonoids can therefore be considered quality markers for differentiating the quality of *D. officinale* stems across the three cultivation environments and may also serve as potential targeted treatments for MAOA and TNF. Moreover, the down-regulated metabolites showed binding activity with SRC and PTGS2, with energy values below -4.25 kcal·mol^-^¹ (except for 5-aminonovaleric acid). Dukunolide A had the lowest docking energy with SRC at -9.8 kcal·mol^-^¹, while lyciumumide A had the lowest docking energy with PTGS2 at -10.6 kcal·mol^-^¹. Eighteen up-regulated flavonoids were selected for molecular docking visualization with MAOA and TNF (**Figure 7**). Similarly, down-regulated metabolites with low binding energies were chosen for molecular docking visualization with SRC and PTGS2 (**Supplementary Figure S7**).

### 3.3 *In vitro* experiments

**Figure 8:**
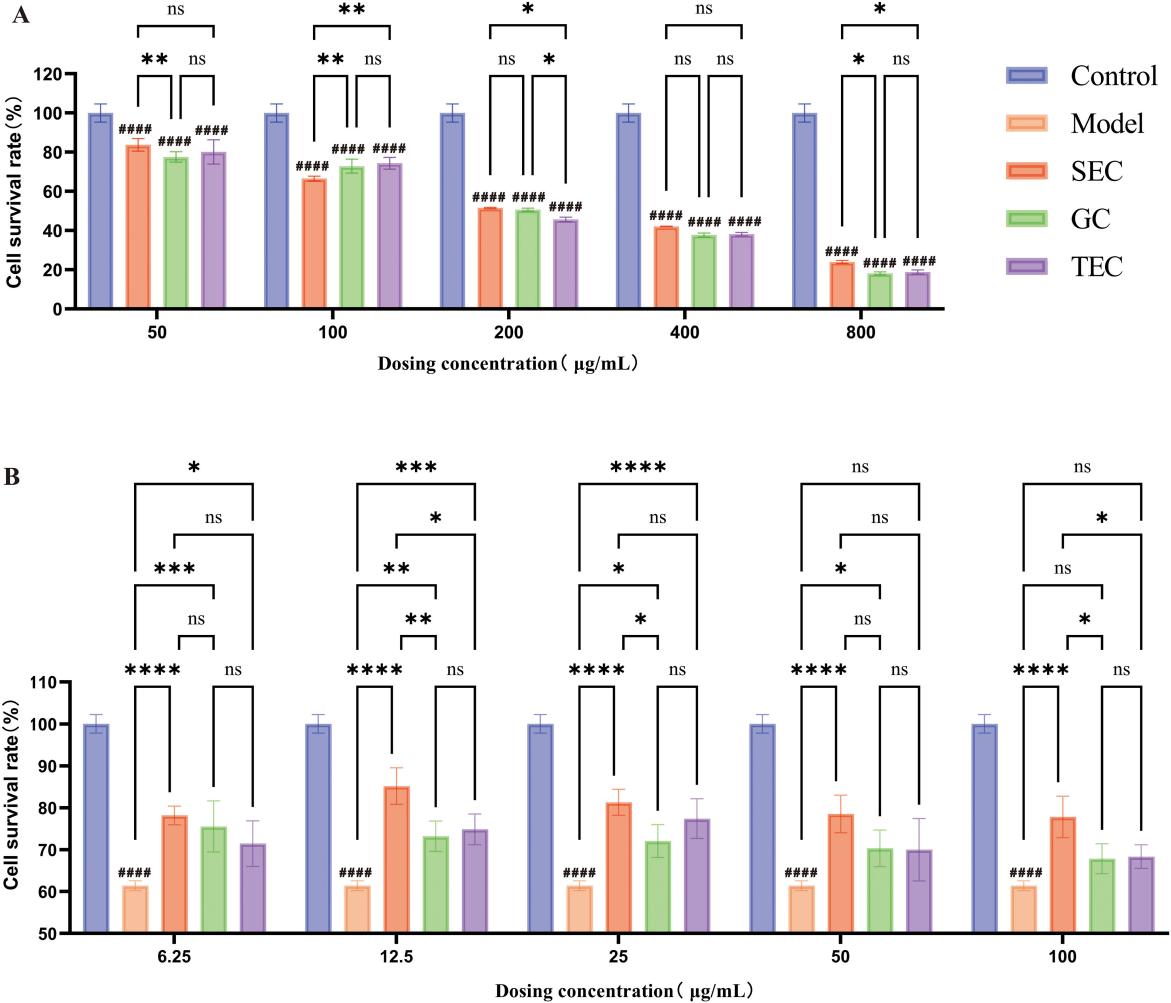
*In vitro* activity results of *D. officinale* stems extract at different concentrations. *p < 0.05; **p < 0.01; ***p < 0.001; ****p < 0.0001; **^####^** *p* < 0.0001, compared with the control group; ns, no significant difference. **(A)** Human liver cancer cell inhibition experiment. **(B)** Cell protection experiment for chronic atrophic gastritis.

To investigate the therapeutic differences of *D. officinale* stems grown in different cultivation environments for liver cancer and chronic atrophic gastritis, this study conducted HepG2 cell inhibition experiments and chronic atrophic gastritis cell protection experiments. The results showed that the inhibitory rate of *D. officinale* stem extracts on liver cancer cells increased with higher concentrations across all cultivation environments (**Figure 8A**). At 100 µg/mL, the SEC extract exhibited stronger inhibitory effects compared to TEC and GC (p < 0.01). However, at 800 µg/mL, SEC performed less effectively than the other two groups (p < 0.05). For other concentrations, the inhibitory effects of the different extracts on cancer cells varied, though SEC did not show a clear advantage overall. The extracts of *D. officinale* stems from different cultivation environments also exhibited protective effects on chronic atrophic gastritis cells. Among all concentration groups, the SEC extracts demonstrated better protective effects compared to TEC and GC (**Figure 8B**), with the highest performance observed at a concentration of 12.5 µg/mL (p < 0.05). This finding aligns with the stomach-benefiting properties outlined in the Chinese Pharmacopoeia (2020 edition).

## 4 Discussion

In this study, a total of 1929 metabolites, including flavonoids, amino acids, alkaloids, lipids, and phenolic acids, were identified in *D. officinale* stems cultivated in three distinct environments, consistent with previous research (Yang et al., 2023b). Differential analysis of metabolites revealed that, compared to the GC and TEC environments, 20 primary metabolites and 36 secondary metabolites were up-regulated in the SEC environment (SEC_vs_other, |Log_2_FC| ≥ 1.0, p < 0.01, VIP > 1). Primary metabolites were mainly enriched in the biosynthesis of amino acids pathway, while secondary metabolites were primarily enriched in the flavone and flavonol biosynthesis pathway. Given that *D. officinale* stems in the SEC environment have long been exposed to high calcium, UV stress, and drought stress in natural settings, previous studies have indicated that kaempferol and quercetin in the flavonoid pathway play vital roles in plant resistance to UV, drought, and salt stress (Sun et al., 2020a; Xu et al., 2020). Additionally, amino acids, essential nutrients for plant growth and development, are critical for plant resistance to drought and other abiotic stresses (Mwadzingeni et al., 2016). Therefore, the enrichment of the flavonoid and amino acid pathways in the SEC environment may be associated with the increased resistance of *D. officinale* stems (cultivated on stones) to UV, drought, and salt stress.

The secondary metabolites up-regulated in the SEC environment were primarily flavonoids (18/36), including biologically active compounds such as homeriocitryol-7-O-β-O-glucoside, phloretin-4’-O-glucoside (phloridzin), nicotiflorin, and isoquercitrin. These compounds exhibit significant therapeutic effects, including in the treatment of acute myocardial infarction (Guan et al., 2000), anti-inflammatory properties (He et al., 2022), prevention of diabetic complications (Lu et al., 2023), antioxidant effects (Juan-Badaturuge et al., 2011), and hypoglycemic activity (Aimudula et al., 2020). Notably, quercetin-3-O-(4’’-O-glucosyl) rhamnoside and rutin were two unique quercetin derivatives found specifically in the SEC environment. Rutin is known for its broad range of physiological and pharmacological activities, including anti-inflammatory (Liu et al., 2022), antidiabetic (Cai et al., 2023), and anticancer properties (Soosai et al., 2024). Additionally, 7 amino acids and their derivatives were found exclusively among the up-regulated primary metabolites in the SEC environment, with L-glutamine being particularly notable for its potential therapeutic effects in treating diabetes (Samocha-Bonet et al., 2011) and gastritis (Ren et al., 2013).

Network pharmacology involves utilizing databases such as proteomics, genomics, and bioinformatics to perform systematic analyses of medicinal plants at both molecular and holistic levels (Liu et al., 2024). In this study, metabolomics was employed to analyze the differential metabolites in *D. officinale* stems cultivated in different environments, and their corresponding targets were identified using databases. Through the construction of PPI networks and weighted analysis, two key up-regulated targets, MAOA and TNF, and two key down-regulated targets, SRC and PTGS2, were identified. To verify the binding capacity between the core targets and differential metabolites, molecular docking was conducted. Notably, flavonoids exhibited superior binding affinity with MAOA and TNF compared to other up-regulated compounds. These findings suggest that the collective action of these 18 flavonoids likely contributes to the stomach-benefiting and anti-inflammatory properties of *D. officinale* stems, highlighting their potential as targeted drugs for MAOA and TNF. Specifically, kaempferol-3-O-(6’’-malonyl) sophorotrioside demonstrated the best binding capacity, while nicotiflorin and isoquercitrin, which are involved in the flavone and flavonol biosynthetic pathway (**Figure 3B**), also displayed excellent binding affinity. Previous studies have shown that nicotiflorin exhibits anti-inflammatory, hypoglycemic, and other biological activities (Juan-Badaturuge et al., 2011; Aimudula et al., 2020), while isoquercitrin can be used to prevent diabetic complications (Lu et al., 2023). Thus, the content of nicotiflorin and isoquercitrin serves as a key quality marker for assessing the therapeutic efficacy of *D. officinale* stems cultivated in different environments.

A study has shown that extracts from *D. officinale* stems can significantly inhibit the growth of human hepatocellular carcinoma cells (HepG2) (Xing et al., 2018). Additionally, granules made from *D. officinale* stems are currently undergoing clinical trials for the treatment of chronic atrophic gastritis (Yang et al., 2023a). To explore the therapeutic differences of *D. officinale* stems cultivated in various environments, this study conducted HepG2 cell inhibition experiments and chronic atrophic gastritis cell protection experiments. Simultaneously, network pharmacology was used to analyze the underlying causes of these differences in therapeutic efficacy. The results revealed that while no significant difference was observed in the inhibitory effects of *D. officinale* stems from different cultivation environments on HepG2 cells (**Figure 8A**), the SEC environment showed superior protective effects on chronic atrophic gastritis cells compared to the TEC and GC environments (**Figure 8B**). The traditional efficacy of *D. officinale* stems, known for nourishing the stomach and promoting the production of body fluid, aligns with our *in vitro* findings (Yang et al., 2023a). MAOA inhibits the metastasis of hepatocellular carcinoma cells by suppressing the adrenergic system and activating the epidermal growth factor receptor (EGFR) signaling pathway, showing a negative correlation with hepatocellular carcinoma progression (Li et al., 2014). TNF, typically referring to TNF-α, is recognized as a key regulatory factor in inflammatory responses (Croft, 2009). Meanwhile, SRC promotes the repair of inflammation-mediated intestinal mucosal damage by promoting cell proliferation and inhibiting apoptosis, while also activating related inflammatory pathways to drive inflammation’s onset and progression (Dong et al., 2023). PTGS2 triggers inflammatory responses via the NF-κB pathway, with its expression, induced by cytokines and growth factors, being up-regulated during inflammation (Kunzmann et al., 2013). In the SEC environment, the up-regulated flavonoids in *D. officinale* stems compete with TNF-α for TNF receptors (Hayden and Ghosh, 2014; Sabio and Davis, 2014). Furthermore, the down-regulated metabolites bind less to SRC and PTGS2, decreasing inflammation production and thereby inhibiting inflammation (Roskoski, 2015). This provides an explanation for the superior protective effects of *D. officinale* stems in the SEC environment on chronic atrophic gastritis cells.

In summary, compared to the TEC and GC environments, the SEC environment exhibited 18 up-regulated flavonoids and 7 amino acids as key differential metabolites. Nicotiflorin and isoquercitrin not only demonstrated excellent binding affinity with the core targets MAOA and TNF, but also possess a broad range of biological activities, making them ideal as core quality markers. *In vitro* experiments further confirmed the superior protective effect of *D. officinale* stems from the SEC environment on cells with chronic atrophic gastritis, with changes in target preference being a crucial factor contributing to the observed differences in efficacy. However, despite the careful design of the experiments in this study, cellular models cannot fully replicate the complex *in vivo* environment of living organisms. Future research should prioritize further pharmacological studies on nicotiflorin and isoquercitrin within the context of *D. officinale* stems from the SEC environment. Such studies, focusing on molecular pharmacology and *in vivo* experiments, will provide vital scientific insights into the medicinal value and quality assessment of *D. officinale* stems cultivated in natural environments.

## 5 Conclusions

In conclusion, this study selected 18 flavonoids as references for cultivating high-quality *D. officinale* stems through targeted metabolomics, weighted network pharmacology, and molecular docking. These flavonoids showed potential as targeted therapies for MAOA and TNF. In addition, modern pharmacological research highlighted nicotiflorin and isoquercitrin as the most important quality markers. *In vitro* experiments confirmed that *D. officinale* stems grown in SEC environment exhibited more significant protective effects on chronic atrophic gastritis cells. This was attributed to the combined effects of enhanced binding of differential metabolites to targets like MAOA and TNF, and reduced binding to targets like SRC and PTGS2. In the future, to fully enhance the medicinal quality of *D. officinale* stems, priority should be given to the development of the *D. officinale* cultivation industry under the SEC environment. Future research should consider conducting further pharmacological studies on the 18 flavonoid compounds, with a focus on molecular pharmacology and *in vivo* experiments. This study suggests that the simulated wild cultivation industry of medicinal plants should be fully developed to improve their quality. A combined approach involving widely targeted metabolomics, network pharmacology, molecular docking, and cellular experiments can be utilized to explore pharmacological differences among other medicinal plants.

## Data Availability

The original contributions presented in the study are included in the article/**Supplementary Material**. Further inquiries can be directed to the corresponding author.

## Supplementary material

Supplementary Table 1 Degree values of differential metabolite targets.

Supplementary Table 2 Metabolomic raw data of *D. officinale* stems in different cultivation environments.

Supplementary Table 3 Fold change of differential metabolites from *D. officinale* stems.

Supplementary Table 4 KEGG pathways and related targets.
