# Supplementary material for: Multidimensional evaluation of quality differences for Dendrobium officinale stems grown under different cultivation environments based on widely targeted metabolomics, network pharmacology, molecular docking, and cell experiments
Source: Front Plant Sci. 2025 Mar 28;16:1501545. doi: 10.3389/fpls.2025.1501545 (PMC11985527; doi:10.3389/fpls.2025.1501545)
Supplement: Supplementary file 5 [file DataSheet1.docx]

**
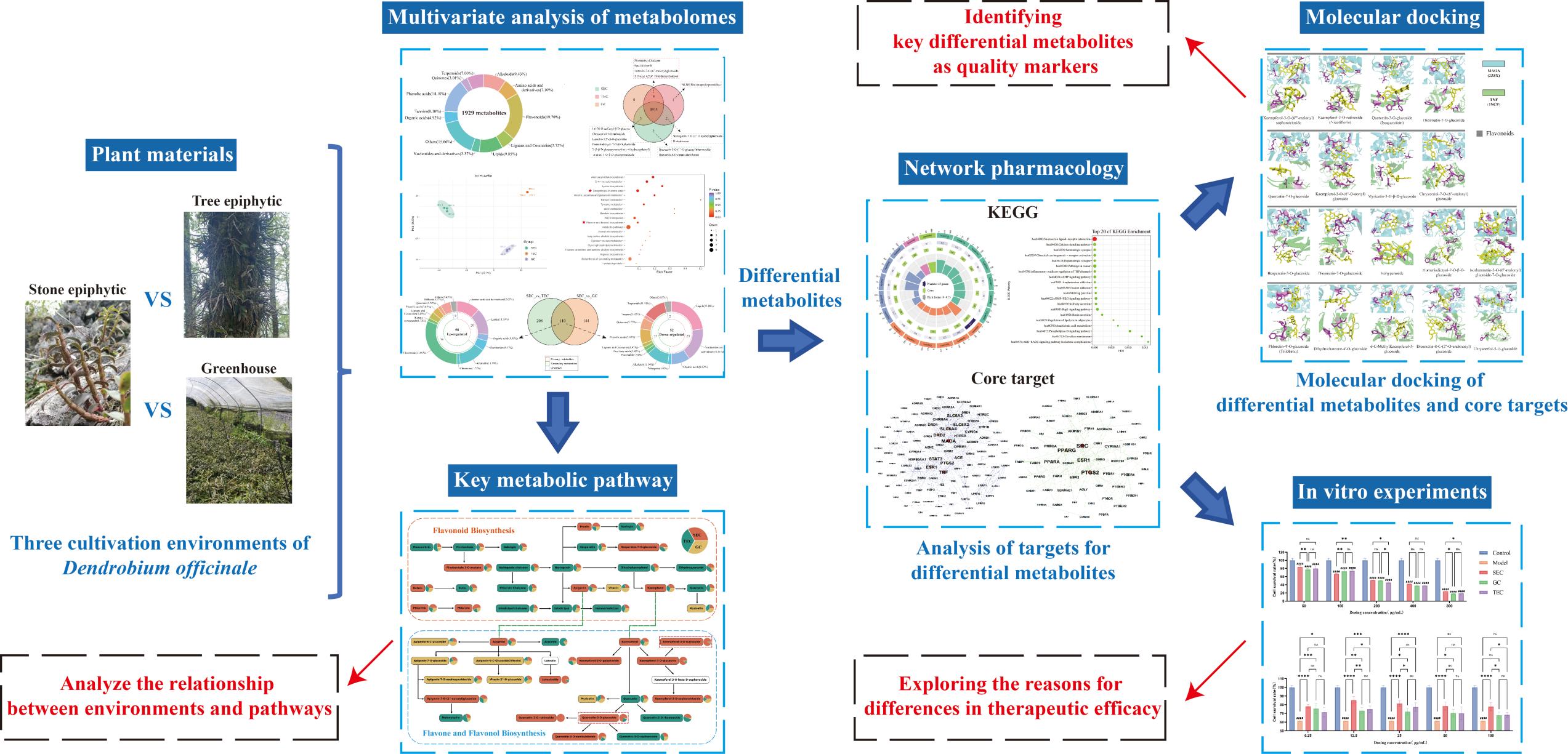
**

**Supplementary Figure S1** Schematic diagram of experimental process.

**
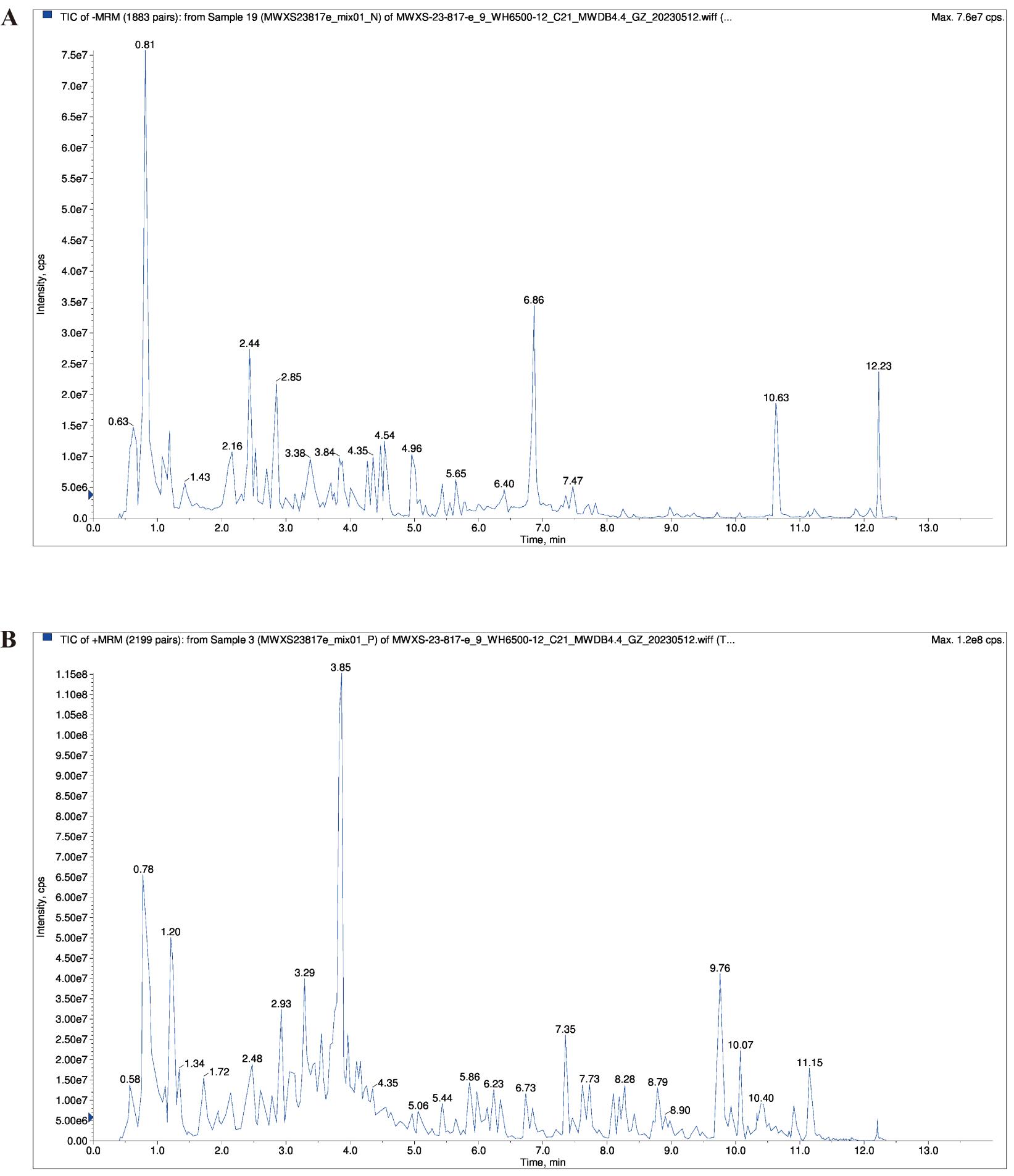
**

**Supplementary Figure S2** Mixed sample mass spectrometry analysis total ion current chart. (A): in negative ion modes. (B): in positive ion modes.


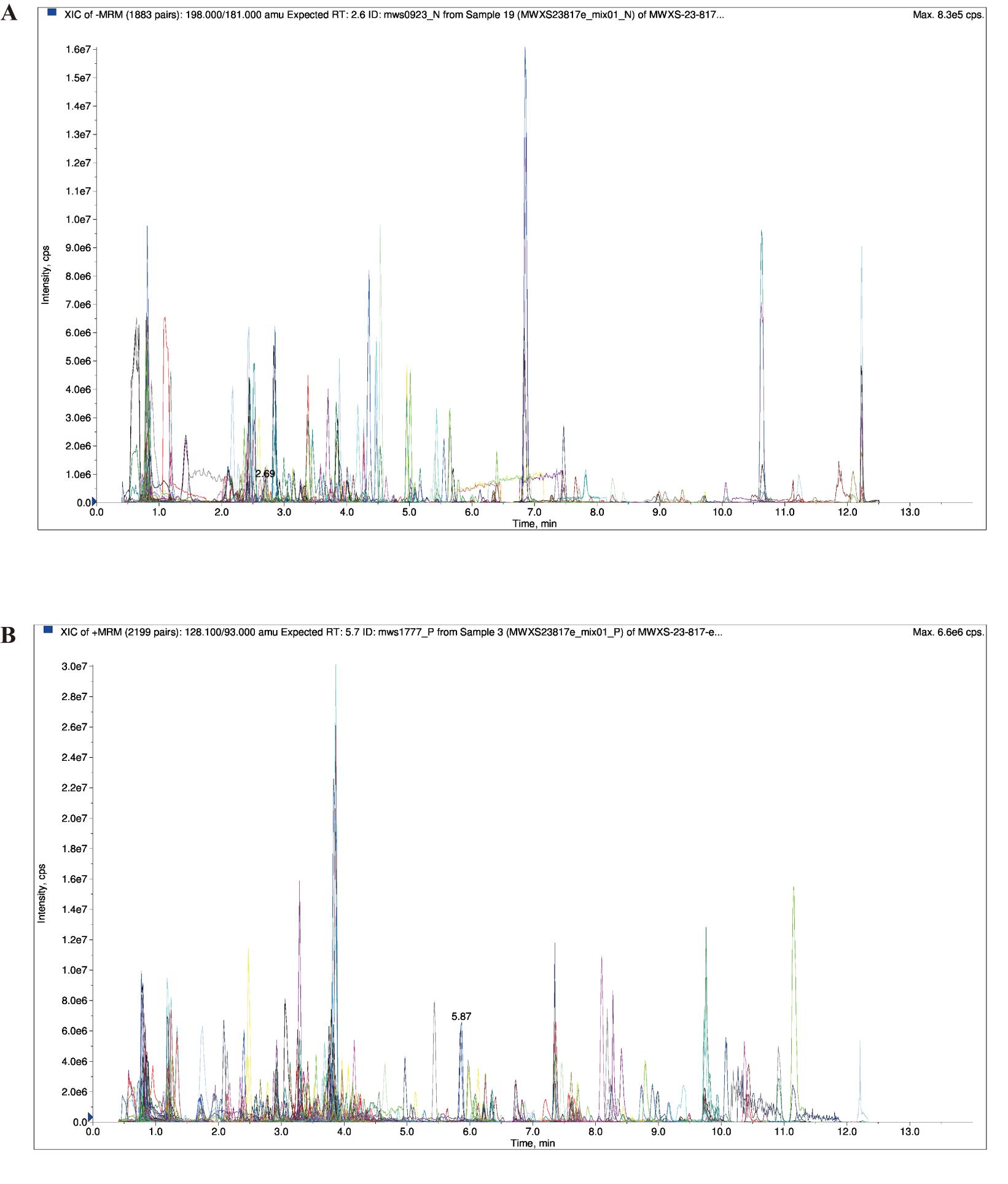
**Supplementary Figure S3** MRM metabolite detection chromatogram. (A): in negative ion modes. (B): in positive ion modes.


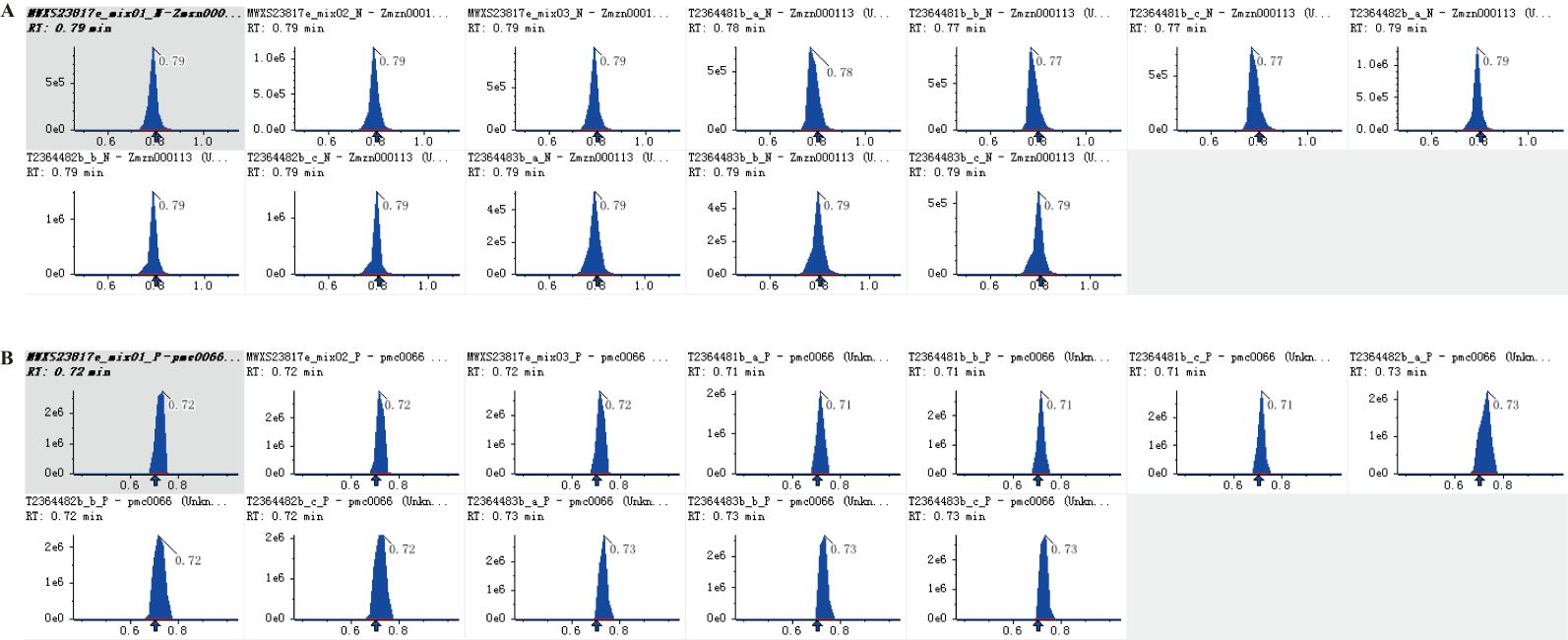


**Supplementary Figure S4** Metabolite quantitative analysis integration correction chart. (A): in negative ion modes. (B): in positive ion modes.

**
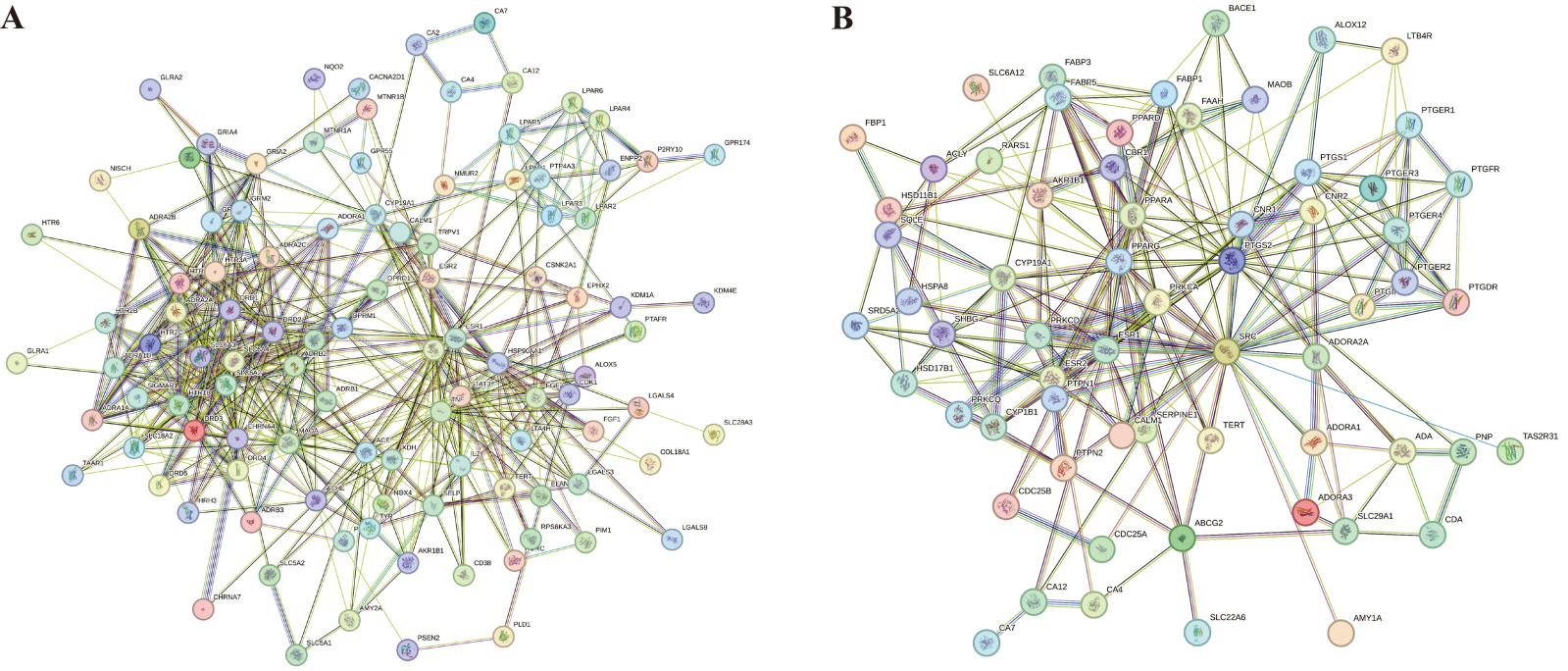
**

**Supplementary Figure S5** Protein interaction network of targets for differential metabolites in *D.officinale*. (A): Up-regulated targets. (B): Down-regulated targets.

**
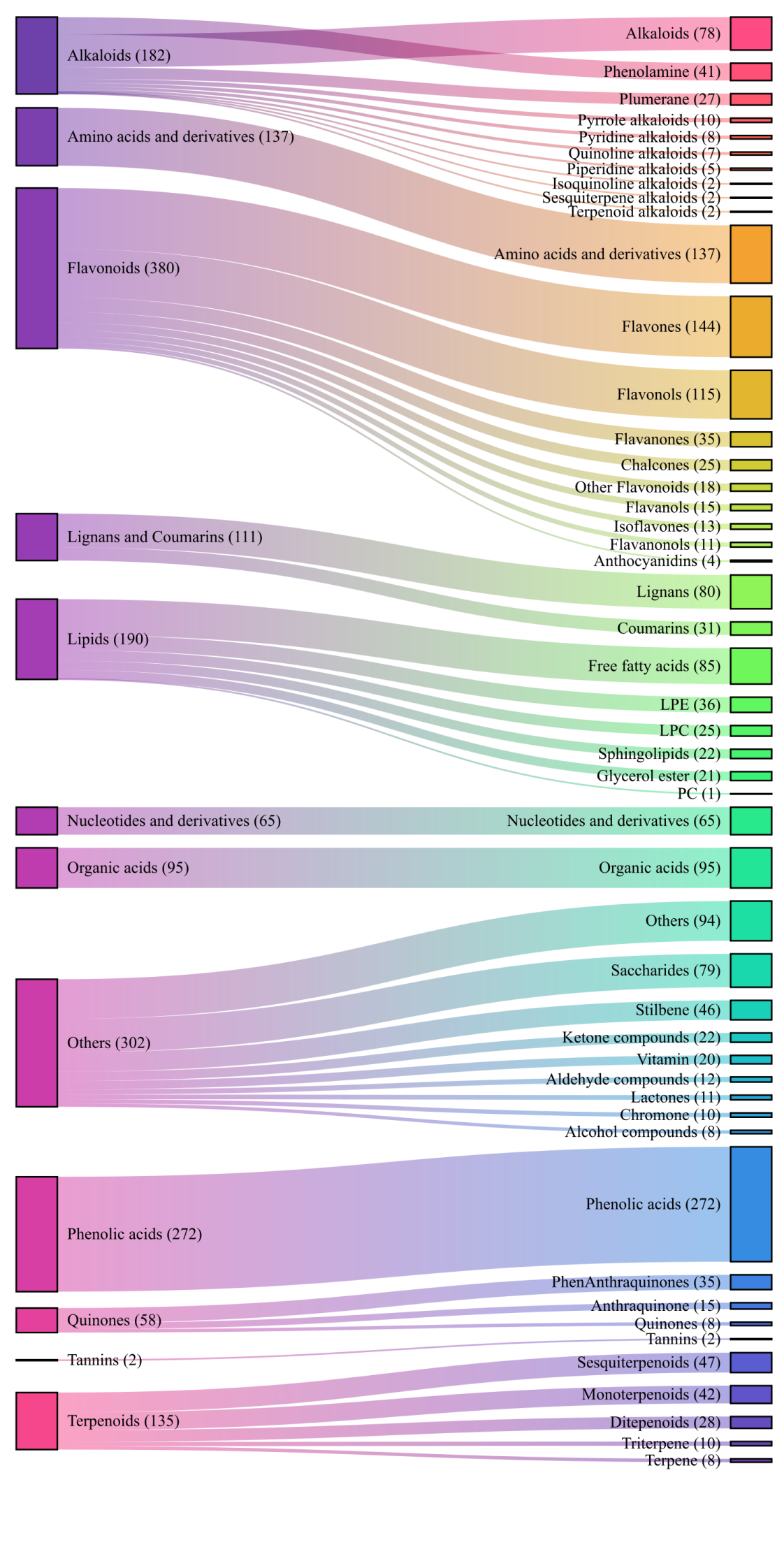
**

**Supplementary Figure S6** Sankey diagram for metabolite classification in *D. officinale* stems.


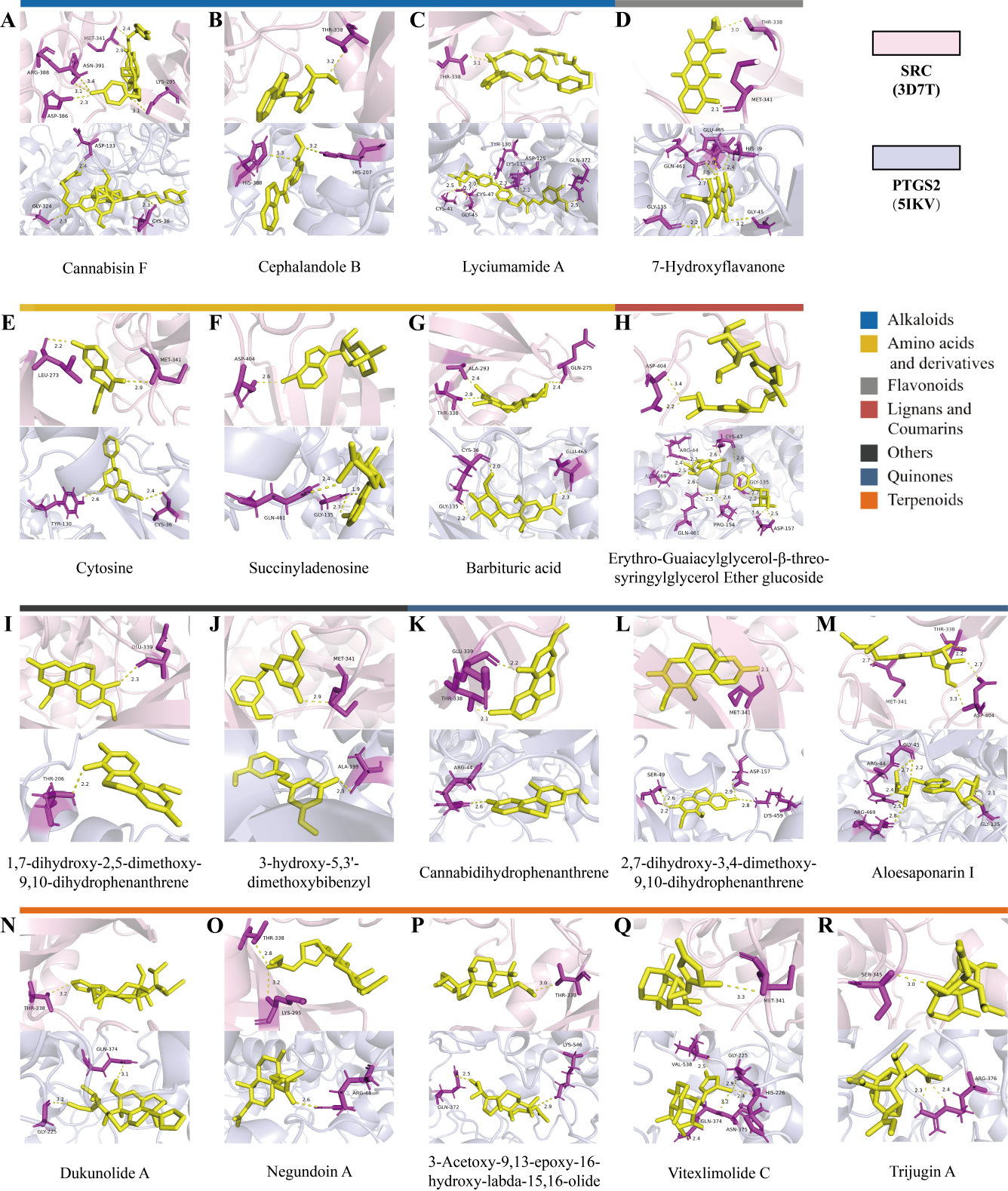


**Supplementary Figure S7** Molecular docking results of down-regulated flavonoids in *D. officinale* stems under different cultivation environments. (A) Predicted binding mode of Cannabisin F with SRC and PTGS2. (B) Predicted binding mode of cephalandole B with SRC and PTGS2. (C) Predicted binding mode of Lyciumamide A with SRC and PTGS2. (D) Predicted binding mode of 7-Hydroxyflavanone with SRC and PTGS2. (E) Predicted binding mode of Cytosine with SRC and PTGS2. (F) Predicted binding mode of Succinyladenosine with SRC and PTGS2. (G) Predicted binding mode of Barbituric acid;Malonylurea;2,4,6-Pyrimidinetrione with SRC and PTGS2. (H) Predicted binding mode of Erythro-Guaiacylglycerol-β-threo-syringylglycerol Ether glucoside with SRC and PTGS2. (I) Predicted binding mode of 1,7-dihydroxy-2,5-dimethoxy-9,10-dihydrophenanthrene with SRC and PTGS2. (J) Predicted binding mode of 3-hydroxy-5,3'-dimethoxybibenzyl with SRC and PTGS2.(K) Predicted binding mode of Cannabidihydrophenanthrene with SRC and PTGS2. (L) Predicted binding mode of 2,7-dihydroxy-3,4-dimethoxy-9,10-dihydrophenanthrene with SRC and PTGS2.(M) Predicted binding mode of Aloesaponarin I with SRC and PTGS2.(N) Predicted binding mode of Dukunolide A with SRC and PTGS2.(O) Predicted binding mode of Negundoin A with SRC and PTGS2.(P) Predicted binding mode of 3-Acetoxy-9,13-epoxy-16-hydroxy-labda-15,16 -olide with SRC and PTGS2.(Q) Predicted binding mode of Vitexlimolide C with SRC and PTGS2.(R) Predicted binding mode of Trijugin A with SRC and PTGS2.
